# Crimean-Congo Hemorrhagic Fever: Tick-Host-Virus Interactions

**DOI:** 10.3389/fcimb.2017.00213

**Published:** 2017-05-26

**Authors:** Anna Papa, Katerina Tsergouli, Katerina Tsioka, Ali Mirazimi

**Affiliations:** ^1^Department of Microbiology, Medical School, Aristotle University of ThessalonikiThessaloniki, Greece; ^2^Department of Clinical Microbiology, Institute for Laboratory Medicine, Karolinska InstituteStockholm, Sweden; ^3^National Veterinary InstituteUppsala, Sweden; ^4^Public Health Agency of SwedenStockholm, Sweden

**Keywords:** Crimean-Congo hemorrhagic fever virus, tick, humans, interactions, immune response

## Abstract

Crimean-Congo hemorrhagic fever virus (CCHFV) is transmitted to humans by bite of infected ticks or by direct contact with blood or tissues of viremic patients or animals. It causes to humans a severe disease with fatality up to 30%. The current knowledge about the vector-host-CCHFV interactions is very limited due to the high-level containment required for CCHFV studies. Among ticks, *Hyalomma* spp. are considered the most competent virus vectors. CCHFV evades the tick immune response, and following its replication in the lining of the tick's midgut, it is disseminated by the hemolymph in the salivary glands and reproductive organs. The introduction of salivary gland secretions into the host cells is the major route via which CCHFV enters the host. Following an initial amplification at the site of inoculation, the virus is spread to the target organs. Apoptosis is induced via both intrinsic and extrinsic pathways. Genetic factors and immune status of the host may affect the release of cytokines which play a major role in disease progression and outcome. It is expected that the use of new technology of metabolomics, transcriptomics and proteomics will lead to improved understanding of CCHFV-host interactions and identify potential targets for blocking the CCHFV transmission.

## Introduction

Crimean-Congo hemorrhagic fever virus (CCHFV, genus Nairovirus, family *Bunyaviridae*) circulates in nature in an enzootic cycle between ticks and non-human vertebrates and poses a significant public health threat due to its high pathogenicity to humans. Humans are infected by bite of infected Ixodid ticks (mainly *Hyalomma* spp.), or by contact with blood or tissues of viremic patients or animals. The disease (CCHF) is characterized by abrupt onset of fever, headache, fatigue, and myalgia, as well as gastrointestinal symptoms, such as nausea, vomiting, and diarrhea. Severe cases present hemorrhagic manifestations ranging from petechiae, epistaxis, ecchymosis, and gingival hemorrhage to severe hemorrhages from various systems. The fatality rate is up to 30%. Wild and domestic animals present a short viremia (2–15 days) and they do not develop clinical illness.

CCHFV is a negative sense, single-stranded RNA virus with a tri-segmented genome consisting of the small (S), medium (M), and large (L) segments which encode the nucleocapsid (N) protein, the glycoprotein precursor (which gives rise to the envelope glycoproteins Gn and Gc) and the L protein, respectively. The CCHFV genome is encapsidated by multiple copies of N protein to form a ribonucleocapsid complex which is critical for the virus replication cycle (Carter et al., 2012). The L protein contains a viral RNA-dependent RNA polymerase domain and an ovarian tumor (OTU) domain with deubiquitinating and deISGylating activities, which is thought to suppress immune signaling (Frias-Staheli et al., 2007).

CCHF endemic foci are present in Africa, Asia, and Europe. Its geographic distribution is associated with that of *Hyalomma* spp. ticks (mainly *H. marginatum, H. rufipes, H. anatolicum*, and *H. asiaticum*) which are the main competent vectors of the virus. The term “vector competence” is used to describe the ability of a vector to acquire, maintain and transmit a pathogen. *H. marginatum* is present in southern Europe and some parts of Asia and Africa. It is characterized by its aggressiveness in seeking human hosts. In CCHF endemic areas, where the climatic and environmental factors are suitable for *H. marginatum* ticks (and their animal hosts), their population is increased in spring and summer, accounting for >30% of tick species in the area. CCHFV has been detected or isolated from additional tick species, but studies are needed to show whether they are competent virus vectors, or merely coincidental unmaintained tick infection from recent feeding on an infected animal or co-feeding (feeding on an uninfected vertebrate host in close proximity with an infected tick) or the result of a recent blood meal on an infected animal. For an arthropod to be incriminated as an actual vector, several criteria must be met; such as vector competence in laboratory studies, and evidence that the arthropod species feeds in nature on a host that develops an appropriate viremia and that it is active at the time of the year that viral transmission is occurring (Reeves, 1957; Turell, 2007). The virus persists in ticks for the duration of the tick lifespan, while the overwintering of the infected ticks plays a critical role in the maintenance of epidemic foci.

As in most arboviral infections, the main players in CCHF are the vector, the pathogen and the host, resulting in the infection (or not) of the host. The co-evolution of the ticks, hosts and pathogens results in conflict or cooperation between them, benefiting ticks and pathogens and, to a lesser extent, hosts (de la Fuente et al., 2016). CCHFV-infected humans may present asymptomatic, mild, severe, or even fatal disease. The course and the outcome of the disease depend on the individual characteristics of the vector, the virus strain and the host, but also on the vector -pathogen-host interactions. The laboratory studies about these interactions are limited due to the high-level containment required for CCHFV and the lack of an animal model, until recently. In this review we will examine the recent findings on CCHFV and discuss the potential contribution of the new technologies to future research in order to better understand the molecular and cellular basis of these interactions.

## Tick-pathogen interactions

**Figure 1:**
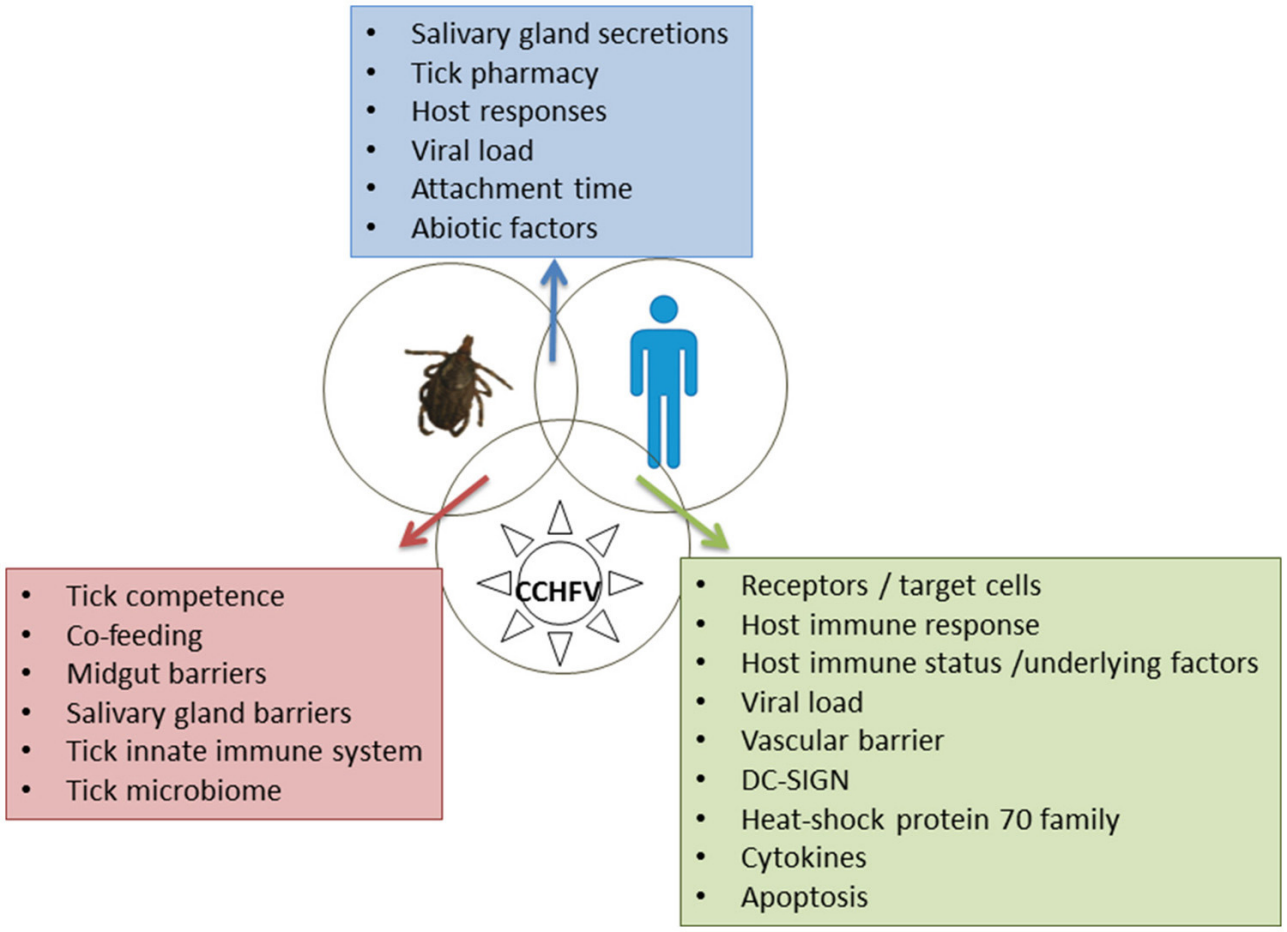
**Tick-host-CCHFV interactions**. Factors playing a major role are shown in the boxes.

Ticks serve as vectors and reservoirs of CCHFV which can be maintained by transovarial and transstadial (from larva to nymph and adult), and, less efficiently, by venereal transmission (Gonzalez et al., 1992). Ixodid ticks, particularly members of the *Hyalomma* genus, are considered main competent vectors, while additional tick species may maintain the enzootic foci of CCHFV circulation between ticks and wild and domestic mammals (Hoogstraal, 1979). The virus has to overcome the midgut and the salivary gland barriers in the tick body (Figure 1). Tick vector competence is influenced by the ability of transmitted pathogens to evade tick innate immune response (Hajdusek et al., 2013).

The molecular events at the tick-pathogen interface are not known. Most likely, the first step is the interaction of CCHFV envelope glycoproteins and the epithelial cells of the ticks. The glycoprotein Gc was shown to be a class II viral fusion protein (Garry and Garry, 2004). Like other invertebrates, ticks do not present adaptive immunity, and they rely on innate immune response consisting of phagocytosis, encapsulation, nodulation, and secretion of humoral factors in the hemolymph (McNally and Bloom, 2014). An additional important mechanism of innate antiviral defense of arthropods (including ticks) against arboviruses, RNA interference (RNAi), was investigated on Hazara nairovirus, which is considered as a surrogate CCHFV model. It was shown that small interfering RNAs (siRNAs), targeting Hazara nairovirus N protein mRNA, inhibited virus replication, and the antiviral effect was stronger when siRNAs were combined with ribavirin (Flusin et al., 2011). The exact role of RNAi in tick-CCHFV interactions remains to be elucidated.

Following a blood meal, CCHFV evades the tick humoral and cellular immune responses and replicates in the lining of the tick's midgut; then it is disseminated to the hemolymph and infects various tissues, with highest viral titers being observed in the proliferating tissues (e.g., salivary glands and reproductive tissues) (Dickson and Turell, 1992). The minimum virus titer necessary to infect the ticks varies among tick species (Shepherd et al., 1991). Following intracoelomic inoculation of CCHFV, virus titer is not affected by tick's sex and feeding status (unfed or engorged), but it is positively related with blood feeding (Dickson and Turell, 1992). CCHFV replication in tissues of an infected tick may be stimulated by tick attachment and feeding on a susceptible host, probably by reducing the stress on a tick induced by viral replication while the tick is waiting to find a vertebrate host, but increase the potential for viral transmission once a host had been acquired (Turell, 2007).

Using a transmission model for CCHFV and next generation sequencing it was shown that many mutations in CCHFV were recovered from ticks after only a single transstadial transmission, whereas no mutations were detected in CCHFV recovered from the mammalian host, with greater viral intra-host diversity in the tick rather than the vertebrate host (Xia et al., 2016).

CCHFV is generally not the sole microbe in ticks; endosymbionts and several pathogens may be present at the same time (Papa et al., 2017). Metagenomic studies showed that the microbiome has an effect on tick fitness and pathogen infection and transmission. As an example, *Francisella*-like endosymbionts have been detected in *Hyalomma* spp. ticks (Ivanov et al., 2011; Szigeti et al., 2014). Although the presence of additional pathogens or endosymbionts may affect the physiology and immune response of the ticks, there are no related studies.

Viral infections in ticks are not entirely silent and may affect the tick survival, behavior and gene expression (McNally and Bloom, 2014). Next generation sequencing of infected and uninfected ticks microbiome may give more insights into the interactions between pathogens and ticks.

## Tick-host interactions

The general issues concerning tick-host interactions likely apply to CCHFV. The first contact between the tick and the host occurs during the tick bite and the prolonged complex process of the tick feeding on the host. The introduction of salivary gland secretions into the feeding lesion is the major, if not exclusive, route via which pathogens and toxins access the vertebrate host and mediate the host reactions (Kaufman, 1989). Despite the host's hemostatic, inflammatory and immune responses, the tick manages to remain attached for blood-feeding via the pharmacy located in its salivary glands and secreted in saliva. Anticoagulants, cytolytic substances, vasoactive mediatiors (such as prostaglandins) and cement, which anchors the mouthparts to the skin, are among the secreted agents. Saliva activated transmission, subsequently renamed saliva-assisted transmission (SAT), affects the host in ways that are exploited by many pathogens to facilitate infection (Nuttall, 1999); SAT is thought to play an additional critical role facilitating the infection of uninfected ticks feeding at the same time on the same host in the absence of an overt host viremia (co-feeding or mechanical transmission) (Gordon et al., 1993). There are no reports on the role of SAT on CCHFV. Because the salivary glands are the most important route for pathogen transmission by arthropod vectors, it is expected that the volume of saliva secreted into the host would be a major factor determining the efficacy of transmission (Kaufman, 2010). Time of attachment may also affect the level of tick-host interaction. Abiotic (environmental and climatic) factors are involved indirectly in the tick-host interactions by playing a role in the abundance and aggressiveness of ticks, thus affecting the chance of a host to be bitten by ticks (Figure 1).

## Host-pathogen interactions

CCHFV must overcome the epithelium and preferentially escape at the basolateral membrane of epithelial cells to establish infection (Connolly-Andersen et al., 2007). CCHFV replicates to high titers at the site of inoculation, in epithelial cells, dendritic cells, and tissue resident macrophages. The productive infection of these cells facilitates spread of the virus and results in early infection of local lymph nodes and peripheral blood-borne monocytes supporting systematic spread of the virus (Burt et al., 1997; Connolly-Andersen et al., 2009; Akinci et al., 2013).

To date the receptor of CCHFV in target cells is not known. The viral glycoproteins Gn and/or Gc are involved in the initial attachment of CCHFV to the cell plasma membrane. It was suggested that Gc is responsible for binding to the cellular receptors, and mediates fusion later, during the early step of replication cycle. An interaction between CCHFV glycoproteins and cell surface nucleolin, a protein found predominantly within nucleoli, has been suggested as putative entry factor; however, more investigations are needed to support the involvement of nucleolin in CCHFV internalization (Xiao et al., 2011). CCHFV enters the cells using clathrin- and the clathrin pit adaptor protein-2 complex, but not caveolin-1 (Simon et al., 2009a; Garrison et al., 2013). Internalization is cholesterol- and pH-dependent (Simon et al., 2009b). Then, CCHFV particles are transported to early endosomes and to multivesicular bodies where the fusion of the virus envelope with cellular membranes takes place. These processes use components of the endosomal sorting complex required for transport regulators (Shtanko et al., 2014).

Cytoskeleton components, including microtubulin and actin filaments, are essential for CCHFV internalization, replication and progeny virus production (Andersson et al., 2004; Simon et al., 2009a). The predicted actin-interacting domain is localized within the central stalk region of the CCHFV N protein adjacent to the coiled-coil motif. The key residue responsible for N protein-actin interaction, D219, and is also crucial for self-association of the N protein (Levingston Macleod et al., 2015). Furthermore, it has been recently shown that the CCHFV N protein interacts with cellular chaperones of the heat shock protein 70 family (including actin), which, in association with DnaJ cofactor adapter proteins, play roles that relate to correct folding and transport of newly synthesized and misfolded proteins and to the assembly of multicomponent complexes (Surtees et al., 2016). One other protein which has been recently demonstrated to be involved in CCHFV replication is aquaporin 6, a water channel that facilitates fluxes of water and small solutes across membranes (Molinas et al., 2016).

The infection of endothelial cells and peripheral blood-borne monocytes results in extravasation into parenchymal tissue enabling the virus to interact with basolateral cells receptors in target organs (Connolly-Andersen et al., 2007). Secondary replication in these organs facilitates the systemic spread of the virus in humans (Akinci et al., 2013). This theory is supported by studies in animal models, which showed that on the first day of infection, the viral replication occurs in the blood, on the second day in spleen and liver, and then spreads systemically to the lungs, kidneys, and brain (Bente et al., 2010).

The virus enters the blood stream overcoming the vascular endothelial surface barrier and the endothelial junctions (Becker et al., 2010). Endothelial cells are targeted either directly by the virus, or indirectly, by virus-induced host-derived soluble mediators that cause endothelial activation (Connolly-Andersen et al., 2011). This has been previously demonstrated for other viral hemorrhagic fevers (Schnittler and Feldmann, 2003). To date, it is not known how CCHFV causes microvascular instability. It is more likely that it is mediated indirectly by increased levels of proinflammatory cytokines, or by a combination of virus infection and the cytokine storm (Connolly-Andersen et al., 2007; Papa et al., 2016).

The endothelial damage is responsible for hemostatic failure by stimulating aggregation and degranulation of the platelets, and activation of the intrinsic coagulation cascade (Weber and Mirazimi, 2008; Bodur et al., 2010). During CCHFV infection, apart from the activated macrophages, an increase in the numbers of natural killer cells and CD3+ CD8+ T cells is observed (Yilmaz et al., 2008; Akinci et al., 2009). But as the disease progresses, the uncontrolled apoptosis of lymphocytes contributes to a depletion in lymphocyte counts, which is presented as lymphopenia (Bente et al., 2010). It has been demonstrated that CCHFV infection can induce apoptosis indirectly, through the release of cytokines from infected cells (Karlberg et al., 2015). This finding fits nicely with the hypothesis described above. Recently it has been shown that CCHFV codes for a non-structural protein, NSs, which may induce apoptosis via both intrinsic and extrinsic pathways (Barnwal et al., 2016).

Soon after the presentation of CCHFV antigen to host cells, innate and adaptive immune responses are activated (Figure 1). DC-SIGN (a calcium-dependent [C-type] lectin cell-surface molecule), which is expressed in the antigen-presenting dendritic cells, was suggested as probable entry factor for CCHFV (Suda et al., 2016). *In vitro* studies showed that RIG-I acts as a pattern recognition receptor for CCHFV and mediates a type I interferon (IFN) antiviral response via the cellular adaptor MAVS (Spengler et al., 2015). As in other viral hemorrhagic fevers, replicating CCHFV delays substantially the IFN response, possibly by interfering with the activation pathway of IRF-3, allowing the rapid viral spread in the host (Andersson et al., 2008). Downregulation of IFN-I signaling pathways relies on the cleavage of ubiquitin and ISG15 from various host proteins (Frias-Staheli et al., 2007). It is of interest that the related CCHFV OTU proteases show clear preferences for ISG15s from certain mammalian species (Deaton et al., 2016).

Several cytokines and chemokines are released during the course of CCHF, especially in severe cases (Ergonul et al., 2006; Papa et al., 2006, 2015, 2016; Saksida et al., 2010). Preliminary analysis showed that the expression of microRNAs related to regulation of cytokine expression is altered in CCHF patients (Demir et al., 2017). Genetic factors and immune status of the host, as well as genetic differences in CCHFV strains, may play a significant role in the virus-host interface, however, there are no studies available.

## Tools for research on tick-host-CCHFV interactions

Tick cell lines are now available to enable the CCHFV studies *in vitro*, offering an alternative approach to understand the way that tick cells respond to virus infection (Bell-Sakyi et al., 2012). The recent development of CCHF virus-like particle (VLP) systems can be used to study cell entry and viral transcription and replication (Devignot et al., 2015; Zivcec et al., 2015). The fact that VLPs are non-infectious will greatly facilitate the tick-pathogen interaction studies under non-BSL-4 conditions. The widespread adaptation of RNA interference (RNAi) will aid in studying tick gene functions (de la Fuente et al., 2005). Interferon response knockout mice have been recently described as animal models for CCHF (Bente et al., 2010; Bereczky et al., 2010; Zivcec et al., 2013). An *in vivo* transmission model for CCHFV in a BSL4 biocontainment was established recently using interferon knockout mice, which is an additional tool to study the transmission and interaction of CCHFV with its tick vector (Gargili et al., 2013). Advances in the study of molecular events at the tick-host-pathogen interface are expected by the increasing number of available genomic resources, including metabolomics, transcriptomics and proteomics. Mathematical and relational models are being constructed for the challenging integration of multi-source datasets from biological systems and cellular networks that would improve our understanding of CCHF pathogenesis (Vidal et al., 2011; Gomez-Cabrero et al., 2014).

## Future perspectives

Over the last decades considerable progress has been made in the identification of the cellular components involved in tick-host-pathogen interactions. However, there is limited knowledge so far in the case of CCHFV due to the high-level containment required for studies with the virus. The identification of the molecular drivers that promote CCHFV survival in the tick, persistence and pathogen transmission provides the opportunity to disrupt these processes and lead to a reduction in tick burden and prevalence of tick-borne diseases (De la Fuente et al., 2017), while the identification of the molecular signaling pathways taking place during the CCHFV-host interactions provides the opportunity to design novel control and vaccine strategies for CCHF. Potential targets could be the cell fusion step during virus entry to the host cells (Garry and Garry, 2004), the pattern recognition receptors for CCHFV (Spengler et al., 2015), the chaperones of the HSP70 family (Surtees et al., 2016), the OTU domain (Frias-Staheli et al., 2007), and the immunogenic factors (Papa et al., 2016). Scientists now have tremendous opportunities to utilize new technologies and *in vitro* models to increase our understanding of CCHFV pathogenesis for the good of Public Health.

## Author contributions

AP wrote the first draft of the article. KTse, KTsi, and AM contributed to the writing of the article. All authors worked for the final version of the article.

### Conflict of interest statement

The authors declare that the research was conducted in the absence of any commercial or financial relationships that could be construed as a potential conflict of interest.
